# Efficacy of applying self-assessment of larviciding operation, Chabahar, Iran

**DOI:** 10.1186/1475-2875-11-329

**Published:** 2012-09-17

**Authors:** Mansour Ranjbar, Khodadad Gorgij, Mahdi Mohammadi, Ali Akbar Haghdoost, Alireza Ansari-Moghaddam, Fatemeh Nikpour, Masoud Salehi, Mohammad Sakeni, Abdolghafar Hasanzahi, Phanthip Olanratmanee, Pattamaporn Kittayapong

**Affiliations:** 1 Center for Vectors and Vector-Borne Diseases and Department of Biology, Mahidol University, Bangkok, Thailand; 2 Health Promotion Research Center, Zahedan University of Medical Sciences, Zahedan, Iran; 3 Physiology Research Center, Kerman University of Medical Sciences, Kerman, Iran; 4 Center for Disease Control and Prevention, Ministry of Health and Medical Education, Tehran, Iran

**Keywords:** Malaria, Larviciding, Self-assessment, Action research, Monitoring, Evaluation

## Abstract

**Background:**

Appropriate supervision, along with availability of an effective system for monitoring and evaluation, is a crucial requirement to guarantee sufficient coverage and quality of malaria vector control procedures. This study evaluated the efficacy of self-assessment practice as a possible innovative method towards achieving high coverage and excellent quality of larviciding operation in Iran.

**Methods:**

The research was conducted on the randomly selected rural health centre of Kanmbel Soliman with 10 staff and 30 villages, in three main steps: (i) assessment of effectiveness of larviciding operations in the study areas before intervention through external assessment by a research team; (ii) self-assessment of larviciding operations (intervention) by staff every quarter for three rounds; and, (iii) determining the effectiveness of applying self-assessment of larviciding operations in the study areas. Two toolkits were used for self-assessment and external evaluation. The impact of self-assessment of larviciding operations was measured by two indicators: percentage of missed breeding habitats and cleaned breeding habitats among randomly selected breeding sites. Moreover, the correlation coefficients were measured between self-assessment measures and scores from external evaluation. The correlation coefficient and Mann Whitney test were used to analyse data.

**Results:**

Following the utilization of self-assessment, the percentage of missed breeding habitats decreased significantly from 14.23% to 1.91% (P <0.001). Additionally, the percentage of cleaned breeding habitats among randomly selected breeding sites increased from 66.89% to 95.28% (P <0.001). The external evaluation also showed significant effects of self-assessment in performance of vector control; the maximum effect of intervention were seen in an action plan for monitoring and evaluation of larviciding operations at field level, geographical reconnaissance for the registration of breeding habitats and worker skills related to larviciding.

Before intervention, the results of self-assessment practice were compatible with external evaluation in 76.3% of 139 reviewed reports of self-assessment. After intervention, the findings of self-assessment and external evaluation were similar in the vast majority of reviewed reports (95%).

**Conclusion:**

The self-assessment tool seems to be valid and reliable in improving effectiveness of larviciding operations. Furthermore, the result of self-assessment is more compatible with external evaluation results if it would be applied frequently. Therefore, it can be used as an alternative assessment technique in the evaluation of larviciding operations in addition to traditional assessment methods.

## Background

Malaria is the world's most important tropical parasitic disease with about 655,000 deaths each year and a further 216 million new cases of malaria diagnosed annually [1-3]. Additionally, more than 3 billion people are now exposed to the risk of acquiring the disease worldwide. It is also the most important parasitic disease in Iran with a total of 1,847 locally-acquired cases and 1,184 imported cases in 2010, of which 10% were *Plasmodium falciparum* and the remaining were *Plasmodium vivax*[2-4]. The south-eastern areas of Iran, including the provinces of Sistan and Baluchestan, Hormozgan and the tropical part of Kerman province, account for around 95% of all malaria cases in the country [5-7].

To achieve the malaria elimination goal in Iran, the Center for Disease Control and Prevention has focused its efforts on the World Health Organization’s (WHO) recommended strategies [8-10], including early detection and proper treatment of malaria cases and the use of preventive measures against the vectors. Indoor residual spraying of insecticide (IRS) and the use of insecticide-treated nets (ITNs) are currently the most commonly used preventive measures against malaria vectors in the global fight against the burden of malaria.

Generally these methods have been effective in the control of *Anopheles* mosquitoes [11-17]. However, the efficacy of IRS is critically affected in some geographical areas of the country due to variation of *Anopheles* species with exophilic and exophagic behaviour [18]. The first biting peak of malaria vectors is at sundown [19] which may possibly decrease the effectiveness of long-lasting insecticide nets (LLINs). Hence, the core interventions of IRS and LLINs could be complemented by other methods such as larval source control in some settings [2]. Accordingly, larviciding has been recommended as one of the main malaria vector control measures, in line with an integrated vector management (IVM) approach [20]. According to WHO reports, larval source management (LSM) is currently considered effective [21] and there is an increasingly tendency for larviciding in the world. Consequently, larviciding has now become a common vector control measure in Eastern Mediterranean Region including Iran as well as South East of Asia in fighting Malaria and Dengue diseases [22].

High coverage and high quality implementation are very important elements in vector control measurements [23]. Strict supervision, along with availability of an effective system for monitoring and evaluation, is a crucial requirement to guarantee sufficient coverage and quality of vector control procedures. There is some evidence that the outcome of larviciding operations in some areas in Iran is not satisfactory and weakness in the monitoring and evaluation of larviciding operations is the main reason for this problem [24].

Some epidemiological studies have shown that self-assessment as a method of monitoring and evaluation is an effective evaluation tool for improvement of organizational/personal performance [11,17,25]. The current study aimed to evaluate the efficacy of self-assessment practice as a possible innovative method towards achieving high coverage and excellent quality of larviciding operations in Iran. It is believed that the developed tools can be useful, not only for the country but also for other nations affected by vector-borne diseases where larviciding is considered as a vector control measure. This is in line with WHO advice, that the development of appropriate tools for monitoring and evaluation of IVM is an operational research priority [23].

## Methods

### Subject selection criteria

Selection criteria for study areas included having a long period of malaria transmission, the prominent role of larviciding in vector control measures, easy access to the field, and willingness of health authorities to use the self-assessment method in larviciding operations. Chabahar was selected as the target district for the present study. Chabahar is located in the south of Sistan and Baluchistan province (south-east Iran) and it is one of the most malarious areas in the country with a more than nine-month transmission season (February to May and July to November).

Among all eight eligible rural health centres (RHC) in Chabahar, Kanmbel Soliman was selected randomly as the study region. Kanmbel Soliman is located in the north of Chabahar district. The 30 villages covered by Kanmbel Soliman RHC have a total population of 5,887 and 1,124 households [26]. *Anopheles stephensi* and *Anopheles culicifacies* have been reported as the main vectors in the study areas [26].

### Design of the study

The study is an action research with a before-and-after approach. It is a triangulation study, combining qualitative study, focus group discussions (FGDs), and quantitative studies; the second part of the study is a quasi-experimental study.

### Data collection methods, instruments used, measurements

Data were gathered by well-qualified data collectors. Validated questionnaires were used to gather expert opinion in order to design the required toolkits. Data regarding effectiveness of larviciding operations and effects of intervention were also collected (using specifically developed toolkits that included questionnaires and recording forms) by direct interviews with related personnel, the review of official documents and field observation.

The study was carried out following three main steps: (i) assessment of the effectiveness of larviciding operations in the study areas before intervention as baseline; (ii) design and establishment of a system for self-assessment of larviciding operations (intervention); and, (iii) determination of the effectiveness of applying self-assessment of larviciding operations in the study areas after intervention.

### Development of toolkits

As a primary phase to facilitate and standardize study procedures two toolkits were developed to determine the effectiveness of larviciding operations (DELT) and for the self-assessment of larviciding operation (SALT). The method for development of the toolkits was as follows:

After investigating similar studies in the other parts of the world [23,27-30] and reviewing available documents on successful monitoring and evaluation of vector control measures that focus on larviciding, questionnaires were devised to collect expert opinions. The questionnaires included a list of related matters, concepts and questions to be used for data collection on DELT and SALT.

Meetings and teleconferences were held between 30 experts and academic staff from international, national, provincial and district levels. After which a final draft of the toolkits was designed using the valuable comments obtained from the above-mentioned specialists in the field of management, monitoring and evaluation of vector-borne diseases. The toolkits and questionnaires were pre-tested in the field and the final versions were developed for this survey.

### Assessment the effectiveness of current larviciding operations in study areas (before intervention)

Using DELT, the effectiveness of larviciding operations before intervention were measured during field visits to 30 villages in Kanmbel Soliman's RHC by the research team acting as external evaluators. Two indicators: “percentage of missed breeding habitats” and “percentage of cleaned breeding habitats among randomly selected breeding sites” were measured as impact indicators of larviciding operations as baseline. The first indicator generally shows the coverage and the second indicator denotes the quality of larviciding operation. Same indicators were measured for determining effectiveness of applying self-assessment of larviciding operation after intervention. In addition, some pertinent processes and output indicators were measured to obtain a clear picture of larviciding operations.

### Percentage of missed breeding habitats

Each village in the study area was searched for open water bodies to determine the percentage of missed breeding habitats (all open bodies of water were taken as potential breeding sites). To locate potential breeding sites, the search was led by malaria experts with a good knowledge of the area. All possible breeding places within houses and other buildings, as well as open spaces within a range of 1.6 km (the average flight radius of local vectors) of human habitation in all 30 target villages, were carefully investigated.

During the external evaluation process all detected breeding habitats were checked by the larviciding operator that they had been registered on the relevant registration sheet and the number of missed breeding habitats (i e, not registered habitats) was determined. the calculation of missed breeding habitats was divided by the total number of detected breeding sites.

### Percentage of cleaned breeding habitats among randomly selected breeding sites

To measure this indicator in villages covered by the study, on average 25% of the total breeding habitats of each village was randomly selected (minimum three, maximum six) and screened for larvae and pupae using a dipper. The assessment was conducted during the transmission season when breeding places were expected to be covered by routine larviciding operations. The number of cleaned breeding habitats was divided by the total number of randomly selected breeding sites to calculate this indicator.

### Method for measuring larvae density in potential breeding sites

The presence of larvae and their density was determined by dipping. From every randomly selected, potential breeding site, up to 10 dips were taken using a standard white 350 ml dipper. A slow-release formulation of *Bacillus thuringiensis* (Bioflash™ granule 10%) was used as the larviciding agent. Based on the product quality control protocol, breeding habitats where no *Anopheles* pupae were recorded by dipping were considered as a cleared site.

### Development of a system for self-assessment of larviciding operation

A system for self-assessment of the operation was designed using lessons learned from other countries and the points of view of local experts and health authorities regarding standard larviciding operations. The system included a toolkit (SALT) and relevant procedures.

### Intervention (establishment of a system for self-assessment of larviciding operations in the target areas)

The research team briefed target groups who were involved in larviciding operations in the study areas regarding the system for self-assessment of larviciding operations and the method of using SALT. Their role and responsibilities, and procedures for the establishment of self-assessment of larviciding operations, were identified by the main investigators. The system had been established in collaboration with local authorities and staff. The researchers advised and supervised local authorities to facilitate implementation of the system. No direct intervention in the procedure of conducting self-assessment of larviciding operations was made by the research team in order to avoid the possibility of any bias in the results.

Using SALT toolkit, self-assessment by the involved staff in larviciding operations was carried out on a quarterly basis. Staff were asked to use the results of their self-assessment to determine areas in need of enhancement and to take necessary action towards the improvement of larviciding operations. Study subjects recorded the results of their self-assessment on the designed forms.

### Determining effectiveness of larviciding operation in the study areas after self-assessment (after intervention)

The effectiveness of applying self-assessment of larviciding operations (after intervention) was measured through field visits to the 30 villages of Kanmbel Soliman's RHC by determining two impact indicators of larviciding operations including “percentage of missed breeding habitats” and “percentage of cleaned breeding habitats among randomly selected breeding sites”. This step was carried out by the research team acting as external evaluators, without giving prior notice to the staff involved in the intervention, exactly one week after the third round of self-assessment. . The method of measuring the indicators was exactly the same as that used in the preceding stage. It was evident that randomly selected breeding habitats for determining cleaned breeding sites were not the same as at the “before” stage. In addition, as in the previous stage (before intervention) the process and output indicators of larviciding operations were measured by the research team to find how larviciding operations worked compared to the previous stage (before intervention) which was considered as the baseline. Therefore, the quantity and quality of changes in the various areas of larviciding operations following the intervention were determined.

### Determining validity of results of self-assessment

To measure validity of the received data from the self-assessment process, the recorded self-assessment reports by staff in the first round, including process and output indicators, were collected by the research team and compared with the results of the first round of the external assessment. In addition, the results of the third round of self-assessment were compared with the results of external assessment that was conducted after intervention. Noting the short period (one week) between the third round of self-assessment and external assessment after intervention, the results were not affected by the time lag between the two evaluations.

### Data management and analysis plan

All indices’ scores were transformed to a scale of 100 and correlation coefficient and Mann Whitney test were used to analyse the data.

### Timeline of the study

Assessing the effectiveness of larviciding operations in the study areas before intervention was conducted from September to October 2010. The system for self-assessment of larviciding operations was established from October 2010 to September 2011. Assessment effectiveness of larviciding operations in the study areas (after intervention) was conducted from September to October 2011.

### Ethical considerations

This study was approved by the Research Ethics Committee of Zahedan University Medical Sciences, Iran. The aims of the project were discussed with all district health authorities and their written or verbal informed consent to cooperate was received. The names of staff pertinent to assessment were kept strictly confidential and were not used in the presentation of the results.

## Results

Table 1 shows the results of larviciding operations before and after establishment of the system for self-assessment of larviciding operations. After intervention and establishment of self-assessment of larviciding operations, the percentage of missed breeding habitats decreased significantly from 14.23% to 1.91% (P <0.001). In comparison, the percentage of cleaned breeding habitats among randomly selected breeding sites increased from 66.89% to 95.28% (P <0.001).

**Table 1:** The parameters of larviciding operations before and after intervention

**Parameter**	**Before**		**After**		**Difference**				**P-value**
	**Mean**	**SD**	**Mean**	**SD**	**Mean**	**SD**	**95%CI**		
							**Lower**	**Upper**	
Percentage of missed breeding habitats	14.23	8.15	1.91	4.59	−12.32	9.60	−15.90	−8.73	< 0.001
Percentage of cleaned breeding habitats among randomly selected breeding sites	66.89	24.82	95.28	9.71	28.39	24.05	19.41	37.37	< 0.001

Based on external assessment, there was a significant increase in all areas of larviciding operations after intervention compared to before. To have a better understanding, all indices’ scores were transformed to a scale of 100 (Table 2). These findings indicate a considerable improvement in the scores of all the evaluated indicators after utilization of self-assessment. The maximum effect of intervention could be seen in having an action plan for monitoring and evaluation of larviciding operations at field level where there is an increase equal to 48.44 in the score compared to before intervention. This has been followed by having a standard geographical reconnaissance for registration of breeding habitats in which the score improved considerably from 50.83 to 94.17. Worker skills related to larviciding came next with an increase of 30.00 after intervention. There has also been some improvement in the remaining two indicators.

**Table 2:** Summary of results of assessment before and after intervention based on external evaluation

**Index**	**Maximum possible Score**	**Before**		**After**		**Difference**				**P-value**
		**Mean**	**SD**	**Mean**	**SD**	**Mean**	**SD**	**95%CI**		
								**Lower**	**Upper**	
EA Field LO action plan	100	56.07	12.72	69.64	9.01	13.57	6.39	7.66	19.48	0.017
EA Worker skills	100	70	8.15	100	0	30	8.15	21.45	38.55	0.027
EA Geographical reconnaissance	100	50.83	7.53	94.17	8.4	43.33	8.98	33.92	52.75	0.027
EA Field supply chain	100	83.5	6.92	98.17	2.48	14.67	7.66	6.63	22.7	0.027
EA Field action Plan for M&E	100	14.07	34.12	62.5	23.15	48.44	22.24	29.85	67.03	0.008

Correlation between total score difference of larviciding operations based on external evaluation before and after intervention and differences of impact of larviciding operations before and after intervention are shown in Table 3. There was a significant negative correlation coefficient between total score difference of larviciding operations after intervention based on external evaluation and decreased percentage of missed breeding habitats after intervention (r = −0.83, P = 0.042). However, the correlation coefficient between total score difference of larviciding operations and increased percentage of cleaned breeding habitats among randomly selected breeding sites after intervention was not significant.

**Table 3:** Correlation between self-assessment and external evaluation

		**Diff. percentage of missed breeding habitats after-before**	**Diff. percentage of cleaned breeding habitats among randomly selected breeding sites After-Before**
Diff. total larviciding operation system. Score after- before based on external evaluation	Correlation Coefficient	−0.83	−0.543
	Sig. (2-tailed)	0.042	0.266
	N	6	6

The findings show an acceptable validity of data from self-assessment practice particularly after intervention when larviciding operators had carried out three rounds of self-assessment. Before intervention, the results of self-assessment practice were compatible with external evaluation in 76.3% of 139 reviewed reports of self-assessment. The remainder were mostly (25.0%) overestimated and a few were underestimated (2.87%). After intervention, the consistency in results between self-assessment and external evaluation increased considerably, such that the findings of both methods were similar in a vast majority of reviewed reports (95%). Although, it is expected that larviciding operators may rate their skills better than the reality with a “positive-self” bias. However, the presence of external evaluation in the present study reduced the possibility of this type of bias.

## Discussion

There is sufficient evidence that self-assessment is a reliable assessment technique in both producing consistent evaluation results across tasks and items as well as contributing to higher individual/organizational achievement and improved performance [31-35]. This study, which was conducted among a population of primary health care staff, demonstrated valid and reliable scales for efficacy of self-assessment in evaluation of larviciding operations. The study illustrated the significant positive effects of self-assessment in the performance of a vector control system. The maximum effect of intervention was seen in having an action plan for monitoring and evaluation of larviciding operations at field level, geographical reconnaissance for registration of breeding habitats and worker skills related to larviciding. Accordingly, the percentage of missed breeding habitats reduced significantly after using the self-assessment approach. Furthermore, the fractions of cleaned breeding habitats among randomly selected breeding sites improved considerably.

No study had examined the efficacy of self-assessment in malaria subjects. However, the utilization of self-assessment has been investigated in other topics, including driving [31,32], nursing [33,34] and athletics [35]. These studies revealed advantages, disadvantages and factors that may possibly pave the way to illusory self-assessment of individual skills. Generally, the validity and reliability of self-assessed measurement tools used in these projects were found to be satisfactory. However, there is some evidence of the presence of illusion in all the components examined in this study [31-35]. According to the data, the majority of the studied individuals rated themselves positively on all studied scales regardless of the country. Moreover, there were cross-cultural differences in drivers’ self-assessment [32]. Age and sex were found to be influential factors on self-assessment [32].

The present study supports the findings of these previous studies, claiming that self-assessment provides valid and reliable measures. For example, in the current study the self-assessment measures were significantly correlated with scores of external evaluation and indicated that self-assessment is reliable. Thus, it proves that the self-assessment tool provides valid and reliable measures of larviciding operators’ proficiency. It is suggested that self-assessment is a valuable assessment technique in the evaluation of larviciding operations, particularly when finding gaps and resolving detected defects is the main aim of monitoring and evaluation.

A limited number of sample sizes were a restriction to the present study. However, the study included external evaluation along with self-assessment, which allowed the authors to discover the percentage of discrepancy between the two methods. Therefore, it is believed that this study provides valuable findings to be used in malaria vector control.

## Conclusion

In conclusion, self-assessment is a valid and reliable tool for assessing larviciding operations and that can play a positive role in enhancement of larviciding operations resulting in positive effects on impact of operation. It prompts operators to not only be acquainted with and correct possible shortcomings, but also to establish consolidated plans for improvement in coverage and quality of larviciding operations. The assessment may additionally help them to understand the concerns of others, including local and national health authorities. One of the most important lessons learned from this study is the fact that the results of self –assessment is more reliable and valid when this tool would be repeated and applied constantly as a routing practice. Finally, future research in this field should focus on establishing self-assessment practice in other vector-borne diseases.

## Competing interests

The authors declare that they have no competing interests.

## Authors’ contributions

The overall implementation of this study including survey design, designing toolkits and questionnaires, data management and analysis, report writing and manuscript preparation were joint efforts by the authors of this paper. All authors have made extensive contribution to the review and finalization of the manuscript.
